# Sustained Isometric Wrist Flexion and Extension Maximal Voluntary Contractions on Corticospinal Excitability to Forearm Muscles during Low-Intensity Hand-Gripping

**DOI:** 10.3390/brainsci10070445

**Published:** 2020-07-13

**Authors:** Davis A. Forman, Garrick N. Forman, Bernadette A. Murphy, Michael W. R. Holmes

**Affiliations:** 1 Faculty of Science, Ontario Tech University, Oshawa, ON L1G 0C5, Canada; davis.forman@uoit.ca; 2 Faculty of Applied Health Sciences, Brock University, St. Catharines, ON L2S 3A1, Canada; gf16sq@brocku.ca; 3 Faculty of Health Sciences, Ontario Tech University, Oshawa, ON L1G 0C5, Canada; bernadette.murphy@uoit.ca

**Keywords:** transcranial magnetic stimulation, corticospinal excitability, muscle activity, electromyography, fatigue, maximal voluntary contraction, forearm, wrist extension, wrist flexion, isometric

## Abstract

The wrist extensors demonstrate an earlier fatigue onset than the wrist flexors. However, it is currently unclear whether fatigue induces unique changes in muscle activity or corticospinal excitability between these muscle groups. The purpose of this study was to examine how sustained isometric wrist extension/flexion maximal voluntary contractions (MVCs) influence muscle activity and corticospinal excitability of the forearm. Corticospinal excitability to three wrist flexors and three wrist extensors were measured using motor evoked potentials (MEPs) elicited via transcranial magnetic stimulation. Responses were elicited while participants exerted 10% of their maximal handgrip force, before and after a sustained wrist flexion or extension MVC (performed on separate sessions). Post-fatigue measures were collected up to 10-min post-fatigue. Immediately post-fatigue, extensor muscle activity was significantly greater following the wrist flexion fatigue session, although corticospinal excitability (normalized to muscle activity) was greater on the wrist extension day. Responses were largely unchanged in the wrist flexors. However, for the flexor carpi ulnaris, normalized MEP amplitudes were significantly larger following wrist extension fatigue. These findings demonstrate that sustained isometric flexion/extension MVCs result in a complex reorganization of forearm muscle recruitment strategies during hand-gripping. Based on these findings, previously observed corticospinal behaviour following fatigue may not apply when the fatiguing task and measurement task are different.

## 1. Introduction

Although traditionally defined as a reduced capacity to generate muscle force, fatigue is understood to be a symptom in which both physical and cognitive functions may be limited [1,2]. These limitations arise through interactions of perceived fatigability and performance fatigability; perceived fatigability refers to the subjective state of the individual (and thus, involves subjective measures), while performance fatigability is measured through objective laboratory-based assessments characterizing the functional decline of performance [3]. Performance fatigability can manifest experimentally as decreased voluntary activation [4,5,6], decreased contractile speed [7], and complex modulations of neuromuscular pathways [8]. However, most studies examine the consequences of performance fatigability either at rest or within the same motor task that induced fatigue. In certain aspects, the findings of these studies apply well to sports and workplaces, where the same motor task is often performed long after it has induced performance impairments. However, it is less clear how the effects of performance fatigability induced in one motor task can manifest in another which shares similar muscle actions.

Recently, we explored this topic by examining how sustained wrist flexion and extension MVCs influenced dynamic hand-tracking accuracy [9]. In this study, hand-tracking was performed on a three-degrees-of-freedom wrist manipulandum before and after a sustained MVC, which ceased when participants could no longer maintain 25% of their baseline MVC force. It was hypothesized that wrist extensor fatigue would impair wrist joint stability, and subsequently hand-tracking accuracy, more than fatigue of the flexors, given that the wrist extensors contribute more to total wrist joint stability [10]. However, while nearly every accuracy metric worsened following the sustained MVCs, there was surprisingly no difference between the wrist flexion and extension sessions. The absence of differences was attributed to the unique muscle recruitment patterns between the two muscle groups. During isometric wrist flexion, the wrist extensor muscles exhibit high levels of muscle activity [11]. In contrast, the wrist flexors are largely inactive during wrist extension. These patterns are likely the consequence of several factors: (1) the wrist extensors are smaller and mechanically disadvantaged compared to the flexors [12,13,14,15], (2) the wrist extensors possess muscle lines of action that are closer to radial-ulnar deviation than flexion-extension [16], and (3) the extensors provide greater joint stability to the wrist [10]. Consequently, the wrist extensors must function at a higher percentage of maximal activation [11,17], and this is the primary reason why the extensors fatigue more rapidly than the flexors [18]. It was therefore suggested that sustained wrist flexion may have induced fatigue in both muscle groups [9]. Thus, if wrist extensor fatigue does impair wrist joint stability, and subsequently tracking accuracy, that instability would have been present following each fatigue session. Additional measures of performance fatigability were recommended to explore this possibility.

Of the many ways to quantify performance fatigability, two include measures of muscle activity and corticospinal excitability. Following fatiguing contractions, motor unit discharge rates progressively decrease [19,20], which manifests in electromyography (EMG) as reduced median power frequency. To maintain force output in spite of lower frequencies, motor units will typically be recruited in greater numbers (assuming the contraction wasn’t already maximal) [21,22]. The amplitude of the EMG signal will consequently increase [23,24]. Similarly, motor evoked potentials (MEPs), elicited via transcranial magnetic stimulation (TMS), increase in amplitude immediately following sustained contractions before falling below baseline for up to 30 min [25,26]. This initial increase in corticospinal excitability is thought to originate from supraspinal sources, given that spinal excitability decreases during and immediately after sustained contractions [27]. Functionally speaking, elevated supraspinal excitability is thought to act as a compensatory mechanism to ensure adequate voluntary activation of less excitable motoneurons and is likely driven by intracortical pathways [28,29]. Thus, following a sustained contraction, an increase in EMG and/or MEP amplitudes tends to indicate the development of performance fatigability. However, as stated earlier, these findings occur either at rest or during the same motor task that was used to induce fatigue. It is presently unclear how performance fatigability induced in one task (example: isometric wrist exertions) might manifest in a second motor task (example: hand-gripping).

Thus, the purpose of the present study was to examine the influence of sustained isometric wrist flexion and extension MVCs on forearm muscle activity and corticospinal excitability during a low-intensity handgrip task. Our hypotheses were twofold. (1) Given that the wrist extensors provide significant co-contraction during wrist flexion, it was hypothesized that muscle activity and corticospinal excitability of the extensors would increase equally following both fatigue sessions. (2) It was hypothesized that the wrist flexors, which are highly task-dependent and contribute minimally to co-contraction during wrist extension, would exhibit increased muscle activity and corticospinal excitability only following the wrist flexion fatigue session.

## 2. Materials and Methods

### 2.1. Participants

Experimental procedures were approved by the research ethics boards (REB) of Brock University (REB# 18–154) and Ontario Tech University (REB# 15855). Written consent was obtained from all participants prior to the experiment. Fourteen right-handed males (Height: 182.9 ± 9.0 cm; Weight: 83.9 ± 12.5 kg; Age: 24.9 ± 2.5 years) were recruited for this study. Participants were excluded from participation if they had any known neurological impairments or were unfit for vigorous physical activity, screened via a magnetic stimulation safety checklist [30] and a Physical Activity Readiness Questionnaire for Everyone (PAR-Q+; Canadian Society for Exercise Physiology (CSEP)), respectively.

### 2.2. Experimental Setup

**Figure 1:**
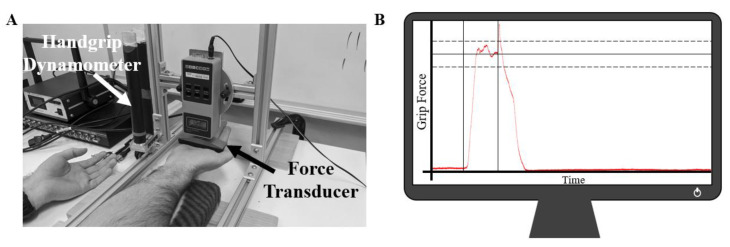
(**A**) Experimental setup for wrist flexion MVCs. In this photo, the force transducer is positioned above the palm and aligned along the distal portion of the metacarpal bones. The forearm could be pronated to perform wrist extension MVCs. The left (non-dominant arm) was rested upon the table, palm up, to prevent any assistance during MVC trials. The same setup was utilized for the fatigue-inducing trial where participants exerted a sustained MVC until they reached the 25% cut-off. The handgrip dynamometer is also shown attached to the aluminum framing in close proximity to the force transducer. (**B**) Example of handgrip force displayed to participants. The solid horizontal line represented 10% of the individual’s maximal handgrip force, while the top and lower dashed lines represented 11 and 9%, respectively. Participants were instructed to begin gripping when the trace reached the first vertical line and would be stimulated 1.5 s later at the second vertical line.

Participants were seated at a table in front of a handgrip dynamometer (MIE Medical Research Ltd., Leeds, UK) suspended above the table top by a custom-built aluminum frame (Figure 1A). With their right elbow supported by a foam pad, participants gripped the handgrip dynamometer with their right hand. Hand placement was established via a band of red tape wrapped around the back prong of the dynamometer. Participants either aligned their index or middle finger (depending on hand size) to the red tape, and this position was maintained throughout the entirety of both collection sessions. The chair was also positioned at a comfortable distance from the table so that participants were neither leaning forward nor overcrowding the table while gripping the handgrip dynamometer. While upper-limb position was not controlled between participants, upper-limb joint angles were manually assessed using a goniometer and matched between the two experimental sessions (elbow extension: 136.0 ± 5.6°; shoulder flexion: 70.1 ± 6.1°; shoulder internal rotation: 2.5 ± 2.2°). In this posture, muscle activity and corticospinal excitability were assessed while participants exerted 10% of their maximal grip force upon the handgrip dynamometer. Hand-gripping was chosen as the measurement motor task over isolated wrist flexion or extension, as hand-gripping is known to elicit muscle activity in *all* forearm muscles (both flexors and extensors) [11,17]. A 10% grip force target was chosen as it is likely comparable to the intensities of many workplaces for continuous work. Grip force was digitally displayed in real-time to participants on a computer monitor (placed directly in front of participants so that they did not have to turn their heads) as a horizontal line representing 10% of their maximal grip force (Figure 1B) (Signal 6, Cambridge Electronic Design Ltd., Cambridge, UK). Two additional horizontal lines (representing 9 and 11% of maximal grip force) were also displayed as an acceptable force margin. During experimental trials, participants were instructed to match the 10% force target as close as possible by squeezing the handgrip dynamometer, but at minimum, to keep their force within the 9 and 11% margins.

All wrist flexion or extension maximal voluntary contractions (MVCs) were performed against a force transducer (Model: BG 500, Mark-10 Corporation, New York, NY, USA) attached to the same aluminum frame as the handgrip dynamometer (Figure 1A). The transducer was raised above the table to allow participants to place their right hand underneath. Two separate foam pads provided support to the forearm during all MVCs; the proximal pad supported the olecranon, while the distal pad supported the distal radio-ulnar joint just proximal to the carpal bones (wrist joint). Two separate pads were utilized over one larger pad in order to allow the forearm surface electrodes (see Electromyography) to be suspended above the table rather than being compressed into the skin during MVCs. For the wrist flexion session, the transducer made contact with the distal anterior surface of the metacarpal bones (top of the palm), while for the wrist extension session, the transducer made contact with the distal posterior surface of the metacarpal bones (back of the most proximal knuckles). This placement for both sessions was marked on the hand with a black marker to match alignment throughout the experiment. For both sessions, the angle of the wrist was maintained at neutral (neither flexed nor extended). While performing MVCs, participants were instructed to (1) rest their left forearm upon the table, supinated, and palm open to limit any assistance from the non-tested limb, (2) keep their right forearm fully in contact with the foam pads at all times (should their forearm come off the pad(s), they were likely using elbow/shoulder flexion to assist in the MVC), (3) maintain an open hand throughout the MVC and to avoid closing their fingers, and (4) to exert maximal force against the transducer by either flexing or extending their wrist (for the wrist flexion or extension sessions, respectively). Participants were provided with ample verbal encouragement from the researchers for all MVCs as well as visual feedback of their force at all times.

### 2.3. Electromyography

Muscle activity was recorded using pairs of surface electrodes (Blue Sensor, Ambu A/S, Ballerup, Denmark) from six muscles of the right arm: flexor carpi radialis (FCR), flexor carpi ulnaris (FCU), flexor digitorum superficialis (FDS), extensor carpi radialis (ECR), extensor carpi ulnaris (ECU), and extensor digitorum communis (EDC). Electrodes were placed over the muscle belly, in-line with fiber orientation, and procedures followed previous placement guidelines [10,11,17,31,32]. A ground electrode was placed on the lateral epicondyle of the right arm. Prior to electrode placement, all recording sites were shaved of hair using a disposable razor and were sanitized with an isopropyl alcohol swab. EMG was band-pass filtered (10–1000 Hz) and differentially amplified (gain of 500; CMRR > 100 dB at 60 Hz; input impedance ~10 GΩ; AMT-8, Bortec Biomedical Ltd., Calgary, AB, Canada). EMG and grip force data were sampled at 5000 Hz (Power 1401–3A, Cambridge Electronic Design Ltd., Cambridge, UK).

### 2.4. TMS

MEPs were elicited in the six forearm muscles via TMS using a Magstim 200 (Magstim, Dyfed, UK). Stimulations were delivered over the participant’s vertex with a circular coil (13.5 cm outside diameter). Vertex was determined by measuring the mid-point between the participant’s nasion and inion and the mid-point between the participant’s tragi. The intersection of these two points was marked and defined as the anatomical vertex of the skull [33]. The coil was held tangentially to the participant’s skull with the direction of the current flow preferentially activating the left motor cortex (thus, activating the individual’s right upper-limb). The coil was held firmly against the participant’s head by one of the investigators to ensure careful and consistent alignment over vertex for each trial. For uniformity, the same experimenter was responsible for holding the coil for all participants and between both collection sessions. Active motor threshold was determined while participants produced 10% of their maximal handgrip force. Active motor threshold was defined as the lowest % of maximum stimulator output (%MSO) that produced a recognizable MEP from the background FDS muscle activity in approximately 4 out of 8 trials [34]. Active motor threshold was determined separately on each collection day and was ultimately not different between the two sessions (Flexion: 41.6 ± 7.1%MSO, Extension: 40.9 ± 5.6%MSO, *p* = 0.44). This stimulation intensity was then increased by 20% and used to elicit MEPs for the remainder of the experimental protocol.

### 2.5. Experimental Protocol

**Figure 2:**
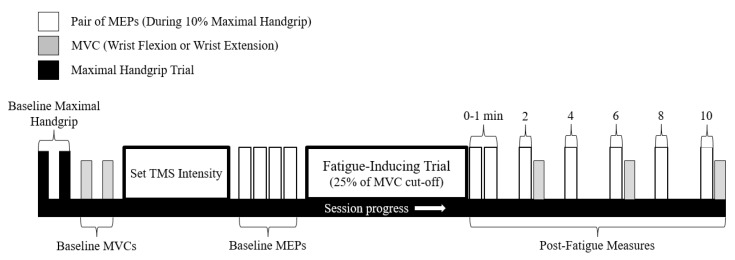
Schematic of the experimental protocol. This protocol was repeated for both the wrist flexion fatigue session and the wrist extension fatigue session (separated by 96 h minimum). The black bars represent the two maximal grip trials performed at the start of the collection. Grey bars represent wrist flexion/extension MVCs (performed on separate days), while white bars represent two MEPs (elicited during 10% of maximal handgrip force).

This experiment consisted of two separate testing sessions. Each session was separated by a minimum of 96 h and consisted of either (1) maximal sustained isometric wrist flexion, or (2) maximal sustained isometric wrist extension (order was pseudorandomized across sample; 8 participants started with flexion, 6 started with extension). A visual overview of the experimental protocol can be seen in Figure 2. Upon obtaining informed consent, participants had electrodes placed over the six forearm muscles of interest (see Electromyography for details) of their right arm. While seated, muscle-specific, isometric maximal voluntary contractions (MVCs) were then performed to determine maximal voluntary excitation (MVE) of all six forearm muscles. MVCs were performed against the manual resistance of one of the researchers and included specific grip and wrist actions to target individual muscle actions (see Table 1 from Forman et al., 2019b). Participants then rotated in their seat to face the custom-built aluminum frame and grasped the attached handgrip-dynamometer. Relevant joint angles of the upper-limb (see Experimental Setup above) were assessed at this time. Participants performed two maximal handgrip trials by squeezing the handgrip dynamometer as hard as possible for 3–5 s. The two trials were separated by one minute of rest, and the higher force value of these two trials was deemed the true maximal handgrip force. Participants then rested their forearm upon the two foam supports and had their right hand aligned to the force transducer, which was raised above the table. Two wrist flexion or extension MVC trials (flexion MVC on the flexion collection session, extension MVC on the extension collection session) were then performed for 3–5 s with one minute of rest provided between trials. The greater of the two trials was deemed their true wrist flexion or extension MVC.

Participants then returned their right hand to the handgrip dynamometer and had their scalp marked for TMS coil placement (see TMS for details). Once marked, participants were familiarized with the computer display and the handgrip force targets (10 ± 1% of maximal handgrip force). Participants were instructed to begin gripping up to and hold the 10% target 1.5 s prior to being stimulated (this time period was marked with two vertical lines; see Figure 1B) and to relax once they had been stimulated. To ensure that participants were sufficiently matching the 10% handgrip force, a number of practice trials were provided prior to any stimulations. The number of trials varied between people (typically 5–10 trials) depending on how quickly they learned to produce 10% of their maximal handgrip. Following sufficient practice, TMS was then delivered over vertex while participants matched the force target. The stimulation intensity began at 30%MSO for all participants and was steadily increased until active motor threshold was established (see TMS for details). This stimulation intensity was then increased by 20% and used throughout the remainder of the protocol. Following the establishment of TMS intensity, 8 baseline MEPs were collected while participants matched the handgrip force target. Each MEP was separated by 15 s to allow for sufficient MEP amplitude recovery [35] and is in following with previous research methods [36,37].

For the fatigue-inducing trial, participants placed their right hand back underneath the table-mounted force transducer. Fatigue was then induced by a maximal sustained isometric wrist flexion/extension (on separate days) MVC. The MVC was performed following the same guidelines as mentioned in Experimental Setup (hand open on both days). The cut-off criteria for the sustained MVC was when participants could no longer maintain 25% of their pre-fatigue MVC force. This cut-off criteria was not disclosed to participants, who were instead told that they would be exerting maximal force for approximately 1–2 min. They were to relax only once the researchers (who were actively monitoring the force readings) told them to stop. Ample verbal encouragement was provided to participants throughout the fatigue-inducing trial.

Following the 25% cut-off, participants immediately returned their right hand to the handgrip dynamometer to perform their first post-fatigue MEP trial. While the time between the end of the fatigue-inducing trial and the start of the first post-fatigue MEP was not measured, it is estimated that the time period was approximately 5 s; since the force transducer and grip dynamometer were attached to the same frame, transition time was minimal. The first MEP trial was labeled as “0” minutes post-fatigue. All MEPs post-fatigue were grouped into pairs and occurred at 0, 0.5, 2, 4, 6, 8, and 10 min (or 0, 30, 120, 240, 360, 480, and 600 s) post-fatigue; the first MEP occurred at the specified time-point, while the second occurred 15 s later. An MVC was performed immediately after the MEP trials at 2, 6, and 10 min post-fatigue to track wrist flexion/extension force recovery (flexion MVCs on flexion fatigue day/extension MVCs on extension fatigue day). These MVCs were not sustained and only lasted approximately 3–5 s.

### 2.6. Data Analysis

Data was analyzed off-line using Signal 6 software (Cambridge Electronic Design Ltd., Cambridge, UK). The peak-to-peak amplitudes of MEPs evoked in all six forearm muscles were individually measured from the initial deflection of the voltage trace from the background muscle activity to the return of the trace to background levels. The amplitudes of the 8 MEPs elicited at baseline were averaged into a single “baseline” value. All MEP amplitudes were then normalized to baseline within each collection session and are shown in figures as “% of baseline”. Mean pre-stimulus handgrip force was measured individually for all MEP trials as a 50 ms window prior to the delivery of stimulation (50 ms prior to the TMS stimulus artifact). Mean Pre-stimulus muscle activity was also assessed in all individual trials for all six forearm muscles. DC offsets were removed, EMG signals were rectified, and the average EMG of a 50 ms window was measured immediately prior to stimulation. The pre-stimulus muscle activity of the 8 baseline handgrip trials were averaged into a single “baseline” value. All pre-stimulus measures of muscle activity were then normalized to baseline within each collection session and are shown in figures as “% of baseline”. Finally, to examine if changes in MEP amplitudes were dictated purely by changes in muscle activity, MEP amplitudes (as a % of baseline) were normalized to muscle activity (as a % of baseline) to make MEP/EMG ratios.

### 2.7. Statistics

Statistical analyses were performed using SPSS software (SPSS, IBM Corporation, Armonk, NY, USA). Assumptions of sphericity were tested with Mauchley’s test of sphericity, and in cases where violated, degrees of freedom were corrected with Greenhouse-Geisser. The assumption of normality was assessed with the Shapiro-Wilk test. A two-way repeated measures ANOVA (fatigue day × measurement time) was conducted for pre-stimulus handgrip force (2 × 15) (% of pre-fatigue maximal grip force) and wrist flexion/extension MVCs (2 × 4/2 × 3; absolute and % change from baseline). To test our main study hypotheses, two separate statistical tests were performed on MEP amplitudes and pre-stimulus EMG: (1) Separate one-way repeated measures ANOVAs (measurement time) were conducted for each collection session on raw data to examine if MEP amplitudes and/or pre-stimulus EMG changed throughout the session (from baseline to post-fatigue measures); (2) A 2 × 14 two-way repeated measures ANOVA (fatigue day × measurement time) was conducted for MEP amplitudes and pre-stimulus EMG normalized to baseline measures (or % change from baseline), as well as MEP/EMG ratios to examine if there were any differences in the post-fatigue period between the two sessions, between any of the post-fatigue measurement times, or an interaction of the two factors. In cases where interactions were observed, separate paired *t*-tests were performed between the two collection sessions at each individual measurement time. In cases where a main effect of measurement time was found, post-hoc pairwise comparisons were conducted with a Bonferroni correction. Effect sizes (ES) were evaluated using partial Eta squared calculated as the division of the sum of squares of the effects (SS_Effect_) by both the SS_Effect_ and the sum of squares of the error (SS_Error_). Significance level was set at *p* < 0.05. Although certain statistical tests were performed on raw MEP and EMG data, all figures are shown normalized to baseline. Group data is reported as mean ± standard deviation (SD) in text and illustrated in figures as standard error (SE).

## 3. Results

### 3.1. Pre-Stimulus Handgrip Force

**Figure 3:**
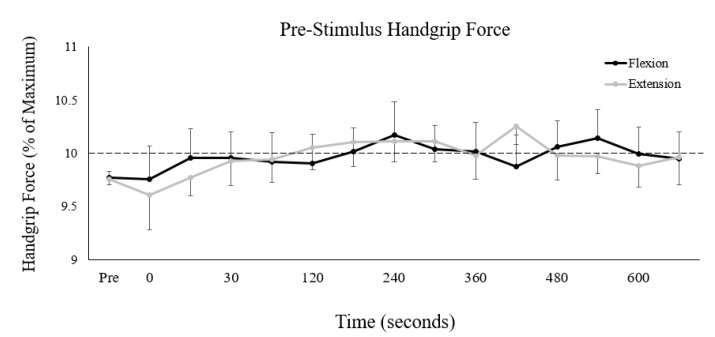
Group averages of pre-stimulus (50 ms) handgrip force immediately prior to TMS. Black/grey lines represent pre-stimulus handgrip force during the flexion/extension fatigue sessions, respectively. The *y*-axis is scaled to the total target range allotted to participants (10 ± 1% of maximal handgrip force). The dashed horizontal line represents the 10% target participants were instructed to match. The *x*-axis displays time of measurement, with “pre” representing the non-fatigued baseline measures and “0” representing the first measurement immediately after the cessation of the fatigue-inducing trial. All unlabeled points occurred 15 s after the preceding time point. Error bars represent standard error.

To compare muscle activity and corticospinal excitability measures throughout this study, it was important that pre-stimulus handgrip force was consistent between the two collection sessions and between pre- and post-fatigue measures (Figure 3).

A 2-way repeated measures ANOVA demonstrated that there was no main effect of collection session (F_(1,13)_ < 0.01, *p* = 0.98, ES < 0.01) or interaction of session and time (F_(14,182)_ = 0.79, *p* = 0.68, ES = 0.06) on pre-stimulus handgrip force. There was, however, a main effect of measurement time on pre-stimulus handgrip force (F_(14,182)_ = 2.04, *p* < 0.05, ES = 0.14), although there were no differences in post-hoc pairwise comparisons between any two measurement times. For group averages, the magnitude of difference was small, and ranged from 9.3–10.3% of maximal handgrip force (a difference of only 1% between the lowest and highest group average values).

### 3.2. Fatigue Inducing Trial

**Figure 4:**
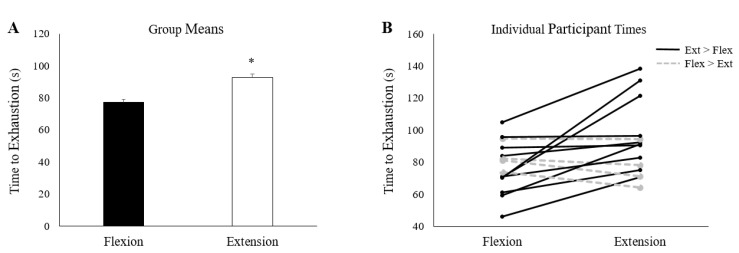
(**A**) Group averages of time to exhaustion. * denotes a significant difference between collection sessions. (**B**) Time to exhaustion for all 14 participants on both testing sessions. Black lines represent the 10 participants who took longer to reach the 25% cut-off on the extension day; grey dashed lines denote the 4 participants who took longer on the flexion day. Error bars in (**A**) represent standard error.

As a group, participants took significantly longer to reach the 25% of pre-fatigue MVC cut-off during wrist extension than they did during wrist flexion (Figure 4A) (Wrist flexion: 77.5 ± 16.0 s, Wrist extension: 92.8 ± 22.8 s, *p* < 0.05). However, there was some variability in this finding; not all participants took longer to reach exhaustion during extension. In total, 10 participants took longer to reach exhaustion during wrist extension, while 4 participants took longer during wrist flexion (Figure 4B).

**Figure 5:**
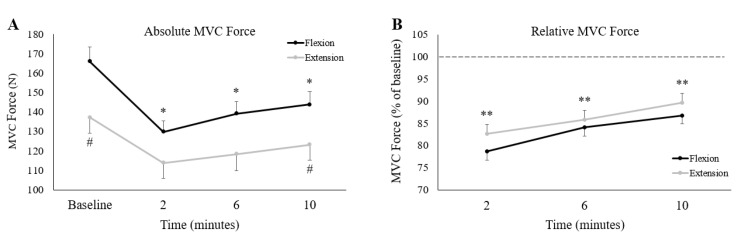
Group averages of (**A**) absolute and (**B**) relative MVC forces between the wrist flexion and extension sessions. Black lines depict wrist flexion MVC force collected on the wrist flexion fatigue day, while grey lines represent wrist extension MVC force collected on the wrist extension fatigue day. The *x*-axis denotes the time of collection; the numbers refer to time of collection after fatigue. The dotted-line in (**B**) represents pre-fatigue (or baseline) MVC force. ^#^ denotes a significant difference between sessions at a single measurement point. * denotes a significant difference of a single measurement point (across both sessions) to baseline. ** denotes a significant difference of a single measurement point (across both sessions) to all other measurement points. Error bars represent standard error.

Prior to fatigue, participants produced significantly more force during wrist flexion than wrist extension (Figure 5A) (wrist flexion: 166 ± 27.2 N, wrist extension: 137.2 ± 28.7 N, *p* < 0.05). There were no significant differences between wrist flexion/extension force throughout the first 6 min following the fatigue-inducing trial, but wrist flexion force was again greater at 10 min post-fatigue (Wrist flexion: 143.8 ± 24.7 N, wrist extension: 123.2 ± 29.1 N, *p* < 0.05). Absolute MVC force significantly decreased following the fatigue-inducing trial and remained significantly reduced from baseline all the way to 10-min post-fatigue (*p* < 0.05 for all three time points). Normalized to baseline MVC force, Figure 5B shows relative MVC force in the post-fatigue recovery period. In this recovery period, there were no significant differences between the flexion or extension sessions (F_(1,12)_ = 0.90, *p* = 0.36, ES = 0.07), nor was there an interaction effect of session and time (F_(2,24)_ = 0.59, *p* = 0.56, ES = 0.05). However, there was a main effect of time (F_(2,24)_ = 28.81, *p* < 0.05, ES = 0.71) with relative MVC forces different from each other at all time points (*p* < 0.05 for all comparisons). This indicates that at each subsequent time point, MVC force was significantly recovering from the previous time point.

### 3.3. Muscle Activity

FCR, FDS, ECR, and EDC all demonstrated a main effect of measurement time on raw (one way ANOVA) pre-stimulus muscle activity (Table 1), although, not necessarily for both testing sessions. The two flexors (FCR and FDS) had a main effect of measurement time only during the extension fatigue sessions, while ECR only demonstrated this effect during the flexion fatigue session. EDC demonstrated a main effect of measurement time for both sessions. Despite these main effects, only ECR showed differences in post-hoc pairwise comparisons. During the flexion fatigue session, ECR pre-stimulus muscle activity significantly increased immediately post-fatigue (time of zero) compared to baseline measures (Baseline: 10.0 ± 3.5 µV, 0: 14.7 ± 5.6 µV, *p* < 0.05). Measurement time had no influence on pre-stimulus muscle activity for FCU or ECU for either fatigue session.

Normalized to baseline, all three wrist extensors demonstrated an interaction effect of session and measurement time on muscle activity (Table 2).

**Figure 6:**
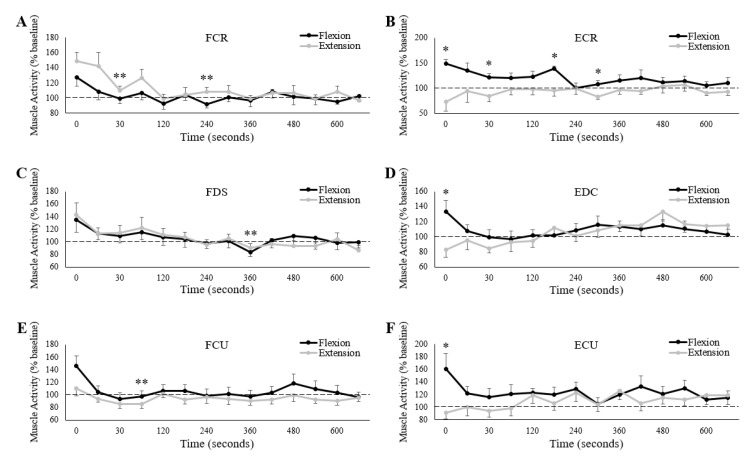
Group averages of normalized (% of baseline) pre-stimulus muscle activity for (**A**) FCR, (**B**) ECR, (**C**) FDS, (**D**) EDC, (**E**) FCU and (**F**) ECU. Black/grey lines are muscle activity collected on the wrist flexion/extension fatigue sessions, respectively. Horizontal dashed lines represent baseline (pre-fatigue) values. The *x*-axes denote time, with “0” representing the first measurement immediately after the cessation of the fatigue-inducing trial. All unlabeled points occurred 15 s after the preceding time point. * denotes a significant difference between sessions at a single measurement point. ** denotes a significant difference of a single measurement point (across both sessions) to “0”. Error bars represent standard error.

Pre-stimulus muscle activity immediately post-fatigue (time point “0”) was significantly greater during the flexion session than the extension session for ECR (Flexion: 149.0 ± 29.5% of baseline, Extension: 73.0 ± 60.9% of baseline, *p* < 0.05), EDC (Flexion: 133.2 ± 53.4% of baseline, Extension: 82.7 ± 36.4% of baseline, *p* < 0.05), and ECU (Flexion: 161.1 ± 78.9% of baseline, Extension: 91.8 ± 31.7% of baseline, *p* < 0.05). For ECR, muscle activity was also greater during the flexion session than extension at 30, 135, and 255 s post-fatigue (*p* < 0.05) (Figure 6B). Separate one-way repeated measures ANOVAs revealed a main effect of measurement time (normalized muscle activity) for ECR during the flexion session and EDC during both sessions. However, only ECR showed any differences in pairwise comparisons from immediately post-fatigue; muscle activity was significantly lower at 600 s post-flexion fatigue (0: 149.0 ± 29.5% of baseline, 600: 106.2 ± 24.0% of baseline, *p* < 0.05).

Although there were no differences in normalized muscle activity between sessions for the three flexors (FCR, FDS, and FCU), all three muscles demonstrated a main effect of measurement time (Table 2). Post-hoc pairwise comparisons also revealed differences from the first measurement point (immediately post-fatigue) for all three muscles. For the FCR, normalized muscle activity was significantly lower at 30 (0: 138.4 ± 42.9% of baseline, 0.5: 105.0 ± 24.6% of baseline, *p* < 0.05), and 240 s (0: 138.4 ± 42.9% of baseline, 240: 100.2 ± 22.4% of baseline, *p* < 0.05) post-fatigue. For the FDS, normalized muscle activity was significantly lower at 360 s post-fatigue (0: 139.4 ± 70.4% of baseline, 360: 86.9 ± 25.6% of baseline, *p* < 0.05), while for the FCU, normalized muscle activity was lower at 45 s post-fatigue (0: 128.2 ± 50.6% of baseline, 45: 100.2 ± 22.4% of baseline, *p* < 0.05).

### 3.4. Corticospinal Excitability

All three wrist flexors, as well as EDC, demonstrated a main effect of measurement time on raw (one way ANOVA) MEP amplitudes (Table 3), although, only FCU was for both testing sessions. FCR and FDS demonstrated a main effect only during the extension fatigue session, while the main effect for EDC only occurred in the flexion session. Despite these main effects, there were no differences in pairwise comparisons between baseline raw MEP amplitudes and any post-fatigue measures.

Normalized to baseline, only FCR demonstrated an interaction effect of session and measurement time on MEP amplitude (Table 4).

**Figure 7:**
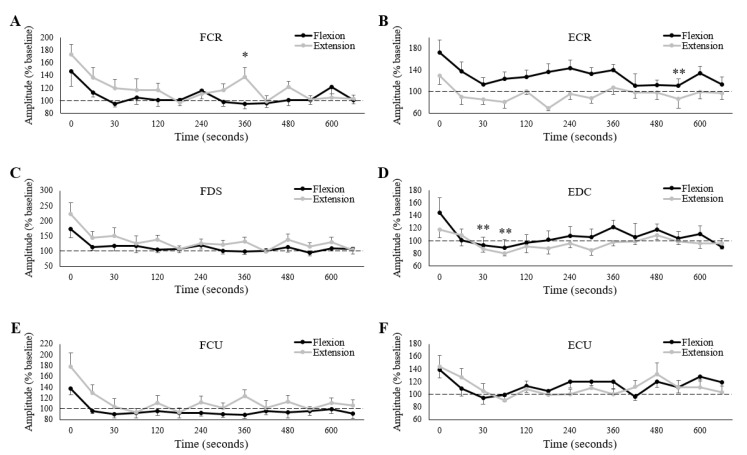
Group averages of normalized (% of baseline) MEP amplitudes for (**A**) FCR, (**B**) ECR, (**C**) FDS, (**D**) EDC, (**E**) FCU and (**F**) ECU. Black/grey lines are muscle activity collected on the wrist flexion/extension fatigue sessions, respectively. Horizontal dashed lines represent baseline (pre-fatigue) values. The *x*-axes denote time, with “0” representing the first measurement immediately after the cessation of the fatigue-inducing trial. All unlabeled points occurred 15 s after the preceding time point. * denotes a significant difference between sessions at a single measurement point. ** denotes a significant difference of a single measurement point (across both sessions) to “0”. Error bars represent standard error.

Separate paired *t*-tests revealed that normalized MEP amplitudes were larger during the extension session than the flexion session 360 s post-fatigue (Figure 7A). Separate one-way repeated measures ANOVAs revealed a main effect of measurement time for both testing sessions, although there were differences in pairwise comparisons only during the extension fatigue session. Compared to immediately post-fatigue, normalized MEP amplitudes were significantly lower at 120 (0: 173.3 ± 58.8% of baseline, 120: 117.4 ± 40.8% of baseline, *p* < 0.05), 135 (0: 173.3 ± 58.8% of baseline, 135: 97.6 ± 23.5% of baseline, *p* < 0.05), and 495 (0: 173.3 ± 58.8% of baseline, 495: 102.3 ± 20.3% of baseline, *p* < 0.05) seconds post-fatigue.

All five remaining muscles demonstrated a main effect of measurement time on normalized MEP amplitudes (Table 4), however, only ECR and EDC showed differences in pair-wise comparisons. Compared to immediately post-fatigue, normalized MEP amplitudes were significantly lower 495 s post-fatigue for ECR (0: 150.9 ± 72.6% of baseline, 495: 99.1 ± 51.3% of baseline, *p* < 0.05) (Figure 7B), and at 30 (0: 131.0 ± 46.8% of baseline, 30: 90.1 ± 20.5% of baseline, *p* < 0.05) and 45 (0: 131.0 ± 46.8% of baseline, 45: 84.8.1 ± 18.4% of baseline, *p* < 0.05) seconds post-fatigue for EDC (Figure 7D). Lastly, ECR was the only forearm muscle that showed a main effect of session (Table 4), with normalized MEP amplitudes significantly larger during the wrist flexion session.

### 3.5. MEP/EMG Ratios

Of the three wrist flexors, only FCU demonstrated a main effect of session on MEP/EMG ratio (Table 5), with ratios significantly larger during the extension fatigue session. Measurement time had no influence on any of the three wrist flexors.

**Figure 8:**
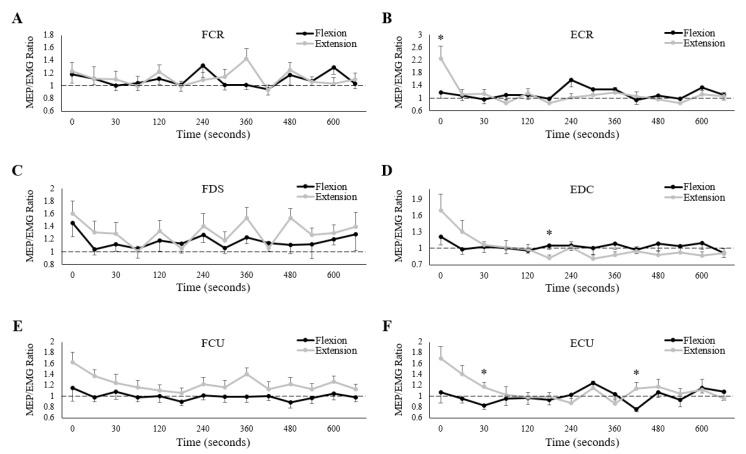
Group averages of MEP/EMG (both values normalized to baseline) ratios for (**A**) FCR, (**B**) ECR, (**C**) FDS, (**D**) EDC, (**E**) FCU and (**F**) ECU. Black/grey lines are muscle activity collected on the wrist flexion/extension fatigue sessions, respectively. Horizontal dashed lines represent baseline (pre-fatigue) values. The *x*-axes denote time, with “0” representing the first measurement immediately after the cessation of the fatigue-inducing trial. All unlabeled points occurred 15 s after the preceding time point. * denotes a significant difference between sessions at a single measurement point. Error bars represent standard error.

For the wrist extensors, all three muscles exhibited interaction effects of session and measurement time on MEP/EMG ratios (Table 5). Separate paired *t*-tests revealed that ratios were significantly larger during the extension fatigue session for ECR immediately post-fatigue (Flexion: 1.17 ± 0.60, Extension: 2.26 ± 1.36, *p* < 0.05) and for ECU at 30 (Flexion: 0.83 ± 0.22, Extension: 1.17 ± 0.29, *p* < 0.05) and 375 s (Flexion: 0.76 ± 0.18, Extension: 1.14 ± 0.35, *p* < 0.05) post-fatigue (Figure 8). For EDC, ratios were larger during the flexion fatigue session at 135 s post-fatigue (Flexion: 1.05 ± 0.25, Extension: 0.83 ± 0.22, *p* < 0.05). Separate one-way repeated measures ANOVAs revealed a main effect of measurement time on MEP/EMG ratios for ECR (both sessions), EDC (only extension), and ECU (only extension), although there were no differences in pairwise comparisons for any muscle.

## 4. Discussion

This is one of the first studies to have examined sustained MVCs performed by opposing muscle groups (wrist flexors/extensors) and their influence on muscle activity and corticospinal excitability. Additionally, this is perhaps the most robust investigation of performance fatigability assessed in a separate motor task (hand-gripping) than what was used to induce fatigue (wrist exertions). While the results demonstrated that performance fatigability changed muscle activity in all forearm muscles to some extent, these adaptations were more complex in the wrist extensors. Corticospinal excitability was also influenced by sustained MVCs, although only FCR and ECR showed differences in normalized MEP amplitudes between sessions. When the changes in MEPs were normalized to the changes in muscle activity, there were notable differences between fatigue sessions. For the wrist extensors in particular, MEP/EMG ratios were significantly larger following wrist extension fatigue, suggesting that supraspinal excitability may have been elevated. These collective results suggest that the effects of performance fatigability may be motor-task specific.

### 4.1. Muscle Activity

As performance fatigability develops, the discharge rate of motor units decreases [19,20,38]. If the sustained contraction is maximal, this reduced firing frequency will result in gradual force and muscle activity loss [39,40]. However, in sustained submaximal contractions, muscle activity can increase if force output remains constant [23,24]. This is made possible by an increase in motor unit recruitment [21,22] that ensures sufficient force production in the presence of more slowly firing motoneurons. Considering these findings, it was hypothesized that (1) wrist extensor muscle activity would increase equally between testing sessions, but also (2) that wrist flexor activity would only increase following sustained wrist flexion. To recap, the wrist extensors contribute more to wrist joint stiffness than the flexors [10]. Consequently, the wrist extensors are more active during isolated wrist flexion than the wrist flexors are during isolated wrist extension [11]. It was therefore anticipated that the wrist extensors would fatigue equally regardless of session. In contrast, the wrist flexors would be mostly inactive during sustained wrist extension.

However, the present findings dispute these hypotheses. There was no difference in muscle activity during hand-gripping between sessions for any of the wrist flexors, while for the wrist extensors, muscle activity was actually greater following sustained wrist flexion than extension. Perhaps most interesting, ECR, EDC, and ECU produced 27.0, 17.3, and 8.2% less muscle activity than baseline, respectively, immediately following sustained wrist extension, although these decreases were not statistically significant (Figure 6B,D,F). This final point begs the question, why did the extensor muscle activity not increase following sustained wrist extension? Unfortunately, drawing comparisons to previous literature is challenging, given that studies typically examine performance fatigability in the same motor task that was used to induce fatigue [8,41]. What can be said is that, during low-intensity hand-gripping, the central nervous system seemingly employs unique control strategies for the wrist flexors and extensors following fatigue of the forearm. Included in this strategy, the activity of the wrist extensors does not increase following sustained wrist extension. Our explanations for this finding are proposed below.

(1) Fatigue specificity: Fatigue was induced by sustained wrist flexion or extension MVCs, but muscle activity was assessed while participants exerted 10% of their maximal handgrip force. While research has demonstrated that muscle activity and certain neurological measures can be state, intensity, and muscle dependent following fatigue [8,42,43], it is intuitive to suggest that they might be task-dependent as well. Not just in terms of the task used to induce performance fatigability but also in terms of the task in which measurements are conducted. For instance, it is possible that handgrip force as low as 10% of maximum can be produced mostly with intrinsic finger muscles–muscles that may not have been fully recruited during maximal wrist exertions. Thus, the muscles that were active during the handgrip task may not have been effectively fatigued during isolated wrist extension. Alternatively, to compensate for this post-fatigue decrease in extensor activity, contributions from other muscles (such as the extensor pollicis longus, which lies deep to the ECR and was not assessed) may have increased. Subsequent investigations utilizing indwelling EMG would add valuable insight to this possibility.
(2) Metabolic optimization: Motor outputs are optimally executed when there is an appropriate balance of joint stability (greatest contribution to joint stability produced by muscle contraction) and metabolic expenditure [44,45]. Prior to fatigue, the level of wrist extensor activity in the present study was theoretically optimal in magnitude and energy expenditure to counter the forces produced by the flexors. However, following wrist extension fatigue, not only would greater motor unit recruitment of the extensors have been needed to exert the same level of co-contraction (since motoneuron discharge rates were likely reduced), but available energy reserves would have also been reduced. Thus, exerting similar baseline forces would cost more energy in a moment of reduced availability. It is therefore possible that wrist joint stability, provided by the wrist extensors, decreased in favour of energy expenditure. While support for this possibility is scarce, some studies have shown that co-contraction [46], limb impedance [47], and joint stiffness [48] all decrease following fatigue. It should be noted that these studies were all conducted during dynamic reaching, not isometric conditions. However, antagonist muscle activity also increases less post-fatigue than agonist activity during isometric actions of the torso [49].
(3) Forearm co-contraction: The suggestions raised above were likely present following the wrist flexion session as well. Thus, it is unclear why extensor muscle activity only increased following the wrist flexion session. Since the wrist flexor muscles demonstrate little activity during isolated wrist extension [11], sustained wrist extension may have only induced fatigue in the wrist extensor muscles. Thus, a feasible reduction in extensor co-contraction (for metabolic purposes) may have been compensated for by other, non-fatigued muscles. In contrast, the wrist extensors are highly active during isolated wrist flexion [11], meaning that performance fatigability was likely induced in the entire forearm following sustained wrist flexion. If so, any reduction in wrist extensor co-contraction might have adversely decreased wrist joint stability. As other forearm muscles were also likely fatigued, and unable to compensate, wrist extensor co-contraction may have increased out of necessity. Thus, muscle activity in all three extensors was higher following sustained wrist flexion than sustained wrist extension.

### 4.2. Corticospinal Excitability

Compared to baseline, resting MEP amplitudes elicited in hand and forearm muscles increase immediately post-fatigue [26,50]. These measures decrease at approximately 15 s and can remain reduced for as long as 30 min [25,26]. It is suspected that the initial increase in corticospinal excitability is due to an increase in supraspinal excitability, given that spinal excitability, measured through motoneuron current injection [51,52] and cervicomedullary evoked potentials (CMEPs) [27], decreases immediately post-fatigue. These findings suggest that spinal excitability is reduced following a sustained contraction but is compensated for by increased supraspinal excitability to ensure adequate motor unit recruitment and volitional drive [8]. The latter half of this statement needs further investigation, given that voluntary activation decreases [4,5,6] and that some measures of cortical inhibition increase following fatigue (see below).

Our hypotheses regarding MEP amplitudes matched our hypotheses for muscle activity, in that post-fatigue MEPs would increase similarly after each session for the wrist extensors, while MEPs would increase to a greater extent for the wrist flexors following wrist flexion fatigue. However, our findings did not support these hypotheses. There was a tendency (only FCR and ECR were significant) for post-fatigue MEP amplitudes to be larger in the wrist flexors following wrist extension (grey dots larger than black; Figure 7A,C,E), while for the wrist extensors (excluding ECU), MEP amplitudes tended to be larger following wrist flexion (black dots larger than grey; Figure 7B,D). Collectively, these results might indicate that corticospinal excitability increases more when forearm muscles are fatigued as the antagonists. However, corticospinal excitability tends to increase with muscle activity [53,54]. In the present study, where the “measurement task” was different from the “fatigue task”, there were complex changes in muscle activity post-fatigue (Figure 6). It was therefore important to consider changes in MEP amplitudes in relation to changes in muscle activity (Figure 8).

When normalized to muscle activity, MEP amplitudes in the extensors tended to be greater immediately following wrist extension fatigue. This suggests that corticospinal excitability was relatively greater on the wrist extension day, and thus, the extensors may have been fatigued to a greater extent than on the wrist flexion day. Counter to our hypothesis, this would indicate that the stabilizing function of the wrist extensors (as antagonists) does not result in equivalent fatigue development compared to agonistic muscle actions. Increased supraspinal excitability almost certainly explains this increase, as spinal/motoneuron excitability decreases following fatigue [55,56,57,58]. The basis for an increase in supraspinal excitability is currently unclear. Given that voluntary activation is reduced following fatigue, it stands to reason that volitional drive, and therefore cortical excitability, must be impaired [8,41]. Literature of underlying mechanisms both supports and opposes this notion. Several studies have shown that cortical inhibition may increase following fatigue. Corticospinal silent periods (CSPs), thought to be indicative of cortical inhibition (via GABA_B_ receptors and inhibitory interneurons [59,60]), increase following fatigue [27,57,61]. EMG suppression, elicited via subthreshold TMS, similarly increases post-fatigue [62], while resting intracortical facilitation (ICF) decreases [29,63]. Additionally, more direct evidence has shown that I waves (repetitive, descending volleys of active pyramidal tract neurons) are reduced in size immediately post-exercise [64]. However, there is also evidence that cortical excitation increases following fatigue. Separate investigations have found no change in ICF following fatigue, while short-interval intracortical inhibition (SICI) decreases following fatigue [28,29,65]. Thus, interpretations of increased supraspinal excitability remain difficult to decipher. It is mostly accepted, however, that said increases indicate the development of fatigue, which occurred in the wrist extensor muscles of the present study.

Interestingly, there were no differences in normalized MEP amplitudes between sessions for either the FCR or FDS; our hypothesis that the wrist flexors would exhibit greater fatigue during the wrist flexion session was therefore not supported. However, in the case of FCU, MEP amplitudes were relatively larger following the wrist extension session (Table 5 and Figure 8E). This finding was surprising, and given known forearm muscle recruitment patterns, it is challenging to explain. One possibility that deserves discussion is the notion of “cross-over” effects of fatigue. While wrist flexion and extension fatigue sessions were performed on separate days to “isolate” the wrist flexors and extensors, respectively, it is difficult to truly isolate a single muscle group while performing a normal human motor task. Not only will the antagonist muscle(s) provide co-contraction during the “isolated” agonist actions, but strong evidence also indicates that performance fatigability can manifest globally [6,66,67]. For instance, fatigue induced in the elbow flexors modulates corticospinal excitability in the contralateral upper-limb [68] and the knee extensors [69]. Similarly, fatigue induced in the knee extensors modulates corticospinal excitability in the elbow flexors [37,70]. These changes are frequently attributed to central mechanisms (possibly increased activity of group III/IV afferents [71]), given a lack of peripheral changes in the non-exercised muscles. Thus, in the present study, it is highly likely that fatigue-inducing wrist flexion modulated the wrist extensors through this cross-over effect, and vice-versa. The surprising responses of the wrist flexors may therefore be the result of not only their functional roles during wrist exertions, but also a cross-over effect of the contracting extensors.

### 4.3. Additional Mechanisms

Since no independent measure of spinal excitability was utilized in this study, attributing modulations in corticospinal excitability to specific sources is limited. That said, alterations in spinal pathways likely occurred to some extent. There is abundant support that muscle spindle discharge rates (which facilitate motoneurons) decrease during sustained efforts [72,73]. This disfacilitation is thought to partially contribute to reduced motoneuron excitability. The behaviour of golgi tendon organs (GTOs) following fatigue is less clear, although some research has shown a reduced sensitivity to passive muscle stretch [74,75]. Intrinsically, motoneuron discharge rates decrease with sustained current injection [51,76], while the motoneuron itself becomes less responsive to afferent and descending excitation [77,78]. These mechanisms contribute to a collective decrease in spinal excitability, and, in studies utilizing stimulation techniques (such as the H-reflex and CMEPs), spinal excitability decreases post-fatigue [55,58,79]. These changes almost certainly influence MEP amplitudes and might exert unique influences between agonist or antagonist fatigue-inducing contractions. Such differences could explain some of the behaviour of forearm muscles in the present study. Future investigations assessing spinal excitability would represent a meaningful improvement on this work.

Lastly, it is mostly accepted that the propriospinal system in humans influences motor outputs by relaying (and subsequently altering) descending and afferent signals to spinal motoneurons [80]. The activity within this system is measurable via stimulation of cutaneous afferents and recording the ensuing suppression of voluntary muscle activity [81]. In the ECR, this suppression increases following sustained wrist extension MVCs but decreases in the coactive triceps brachii [82]. Greater suppression of muscle activity is thought to indicate an increase in inhibition acting upon propriospinal neurons. Thus, propriospinal contributions to descending drive may be impaired following fatigue, at least to the agonist muscle. In the present study, changes to propriospinal activity may have contributed to both corticospinal excitability and muscle activity measures, although further research is needed to confirm this.

### 4.4. Methodological Considerations

TMS intensity was established based solely on the FDS motor threshold. This follows publications in the field that report on multiple muscle sites despite setting stimulation intensities to just one [83,84,85,86]. This is noteworthy for the following reason: while +20% of motor threshold was sufficient to elicit MEPs in all six forearm muscles, motor threshold was found only in FDS. Thus, it is unclear if TMS intensity was 20% greater than the motor thresholds for the remaining five muscles. Small variations in motor thresholds likely exist between muscles, and if so, TMS would have activated slightly different portions of the available motor pool for each muscle. While it is unlikely that this effect fundamentally altered the findings of the present study, it nevertheless deserves mentioning.

Secondly, given the known differences of how maximal versus submaximal contractions influence central [43] and peripheral [42,87] pathways, the results of the present study should not be generalized to lower intensity fatiguing tasks. Likewise, as this study induced fatigue through an isometric contraction, these findings should not be generalized to fatiguing dynamic contractions.

Finally, fatigue was induced while participants maintained an open hand/extended fingers. This was done in the hopes of isolating wrist flexor and extensor muscle contractions. For instance, had a closed hand been used, participants may have squeezed their fist during the wrist extension session. At the very minimum, FDS activity would likely have been high throughout the fatigue-inducing trial, and thus, any differences between sessions might have been difficult to decipher. However, a closed hand is likely more applicable to actions of the workplace. Typically, fatigue of the distal upper-extremity develops while the hand is gripping or manipulating some sort of object. Had a closed hand been used (or should it be used in the future), the results might have differed.

## 5. Conclusions

The purpose of this study was accomplished by examining the influence of sustained isometric wrist flexion and extension MVCs on forearm muscle activity and corticospinal excitability during a separate motor task (low-intensity handgrip). However, the subsequent results supported neither of the main study hypotheses. Sustained wrist flexion and extension did not result in similar increases of wrist extensor muscle activity during hand-gripping (first hypothesis). Rather, a complex adaptation in forearm muscle recruitment was observed, with extensor activity higher as the antagonists. In contrast, fatigue session had no influence on the wrist flexors (second hypothesis). It is possible that a combination of fatigue-specificity and co-contraction optimization contributed to these results. When MEPs were normalized to muscle activity, corticospinal excitability of the wrist extensors was higher following the wrist extension session, while for FCU, responses were surprisingly higher following wrist extension. These increases likely indicate elevated supraspinal excitability, which may be the result of intracortical processes. While possible cross-over effects of fatigue may have contributed to experimental findings, the surprising behaviour of certain forearm muscles remains difficult to explain. The complexity of these results suggest that previous conclusions regarding corticospinal behaviour following fatigue may not apply when the fatiguing task and the measurement task are different. This highlights the need for further research on the specificity of performance fatigability.

## Figures and Tables

**Table 1:** Results from one-way (measurement time) repeated measures ANOVAs performed separately for each collection session on raw pre-stimulus muscle activity. Outputs are listed as *p*-Values, F-statistics, and effect sizes (represented by Partial Eta Squared ($\eta \begin{array}{c} 2\\ p \end{array}$)).

		FCR	FDS	FCU	ECR	EDC	ECU
Flexion	*p*-value	0.54	0.60	0.14	0.02 *	0.01 *	0.07
	F-Statistic	F_(14,182)_ = 0.92	F_(14,182)_ = 0.86	F_(14,182)_ = 1.43	F_(14,182)_ = 2.03	F_(14,182)_ = 2.12	F_(14,182)_ = 1.69
	Effect Size	0.07	0.06	0.12	0.16	0.15	0.16
Extension	*p*-value	<0.001 *	0.02 *	0.32	0.29	<0.001 *	0.19
	F-Statistic	F_(14,182)_ = 3.84	F_(14,182)_ = 1.98	F_(14,182)_ = 1.14	F_(14,182)_ = 1.19	F_(14,182)_ = 3.94	F_(14,182)_ = 1.35
	Effect Size	0.23	0.13	0.09	0.10	0.25	0.13

Notes. * *p* < 0.05.

**Table 2:** Results from two-way repeated measures ANOVAs performed on pre-stimulus muscle activity normalized to baseline.

		FCR	FDS	FCU	ECR	EDC	ECU
Session	*p*-value	0.11	0.97	0.24	0.004 *	0.78	0.10
	F-Statistic	F_(1,13)_ = 3.03	F_(1,13)_ = 0.002	F_(1,11)_ = 1.52	F_(1,11)_ = 13.0	F_(1,12)_ = 0.08	F_(1,9)_ = 3.34
	Effect Size	0.19	<0.001	0.12	0.54	0.01	0.27
Time	*p*-value	<0.001 *	<0.001 *	<0.001 *	0.75	0.001 *	0.40
	F-Statistic	F_(13,169)_ = 4.91	F_(13,169)_ = 4.04	F_(13,143)_ = 3.22	F_(13,143)_ = 0.71	F_(13,156)_ = 2.76	F_(13,117)_ = 1.06
	Effect Size	0.27	0.24	0.23	0.06	0.19	0.11
Interaction	*p*-value	0.51	0.94	0.68	0.014 *	0.003 *	0.02 *
	F-Statistic	F_(13,169)_ = 0.95	F_(13,169)_ = 0.47	F_(13,143)_ = 0.78	F_(13,143)_ = 2.20	F_(13,156)_ = 2.55	F_(13,117)_ = 2.11
	Effect Size	0.07	0.04	0.07	0.16	0.18	0.19

Notes. * *p* < 0.05.

**Table 3:** Results from one-way (measurement time) repeated measures ANOVAs performed separately for each collection session on raw MEP amplitudes.

		FCR	FDS	FCU	ECR	EDC	ECU
Flexion	*p*-value	0.12	0.06	<0.001 *	0.20	<0.001 *	0.12
	F-Statistic	F_(14,182)_ = 1.49	F_(14,182)_ = 1.68	F_(14,154)_ = 3.30	F_(14,154)_ = 1.33	F_(14,168)_ = 3.93	F_(14,140)_ = 1.49
	Effect Size	0.10	0.11	0.23	0.11	0.25	0.13
Extension	*p*-value	<0.001 *	<0.001 *	0.01 *	0.08	0.10	0.14
	F-Statistic	F_(14,182)_ = 3.87	F_(14,182)_ = 3.45	F_(14,154)_ = 2.15	F_(14,154)_ = 1.61	F_(14,168)_ = 1.54	F_(14,140)_ = 1.44
	Effect Size	0.23	0.21	0.16	0.13	0.11	0.13

Notes. * *p* < 0.05.

**Table 4:** Results from two-way repeated measures ANOVAs performed on MEP amplitudes normalized to baseline.

		FCR	FDS	FCU	ECR	EDC	ECU
Session	*p*-value	0.25	0.21	0.12	0.006 *	0.22	0.68
	F-Statistic	F_(1,13)_ = 1.44	F_(1,13)_ = 1.73	F_(1,11)_ = 2.91	F_(1,11)_ = 11.46	F_(1,12)_ = 1.70	F_(1,10)_ = 0.19
	Effect Size	0.10	0.12	0.21	0.51	0.12	0.02
Time	*p*-value	<0.001 *	<0.001 *	<0.001 *	0.001 *	<0.001 *	0.002 *
	F-Statistic	F_(13,169)_ = 5.16	F_(13,169)_ = 5.38	F_(13,143)_ = 5.52	F_(13,143)_ = 2.95	F_(13,156)_ = 4.82	F_(13,130)_ = 2.67
	Effect Size	0.28	0.29	0.33	0.21	0.29	0.21
Interaction	*p*-value	0.04 *	0.41	0.19	0.43	0.58	0.33
	F-Statistic	F_(13,169)_ = 1.84	F_(13,169)_ = 1.05	F_(13,143)_ = 1.35	F_(13,143)_ = 1.03	F_(13,156)_ = 0.88	F_(13,130)_ = 1.14
	Effect Size	0.12	0.07	0.11	0.09	0.07	0.10

Notes. * *p* < 0.05.

**Table 5:** Results from two-way repeated measures ANOVAs performed on ratios of MEP amplitudes (% of baseline) and pre-stimulus muscle activity (% of baseline).

		FCR	FDS	FCU	ECR	EDC	ECU
Session	*p*-value	0.81	0.26	0.02 *	0.90	0.75	0.16
	F-Statistic	F_(1,13)_ = 0.06	F_(1,13)_ = 1.41	F_(1,11)_ = 6.85	F_(1,11)_ = 0.02	F_(1,12)_ = 0.11	F_(1,10)_ = 2.38
	Effect Size	0.01	0.10	0.38	0.002	0.01	0.21
Time	*p*-value	0.18	0.08	0.18	<0.001 *	<0.001 *	0.002 *
	F-Statistic	F_(13,169)_ = 1.55	F_(13,169)_ = 1.95	F_(13,143)_ = 1.70	F_(13,143)_ = 4.12	F_(13,156)_ = 3.43	F_(13,130)_ = 2.71
	Effect Size	0.11	0.13	0.13	0.27	0.22	0.23
Interaction	*p*-value	0.21	0.94	0.80	<0.001 *	0.012 *	0.004 *
	F-Statistic	F_(13,169)_ = 1.47	F_(13,169)_ = 0.46	F_(13,143)_ = 0.66	F_(13,143)_ = 3.95	F_(13,156)_ = 2.20	F_(13,130)_ = 2.56
	Effect Size	0.10	0.03	0.06	0.26	0.16	0.22

Notes. * *p* < 0.05.
